# 

**Published:** 1892-07

**Authors:** 


### UNITED STATES VETERINARY MEDICAL ASSOCIATION.

Arrangements for the annual meeting of the United States Association in Boston September next, are rapidly approaching completion. The meeting promises to be the most important in the history of the organization. An unusual number of instructive features and special arrangements for the entertainment of the members have been provided for.

This is the first three days' session in the history of the Association. The meetıng commences September 20th, and lasts through the 21st and 22d

A cordial reception has been assured to all of the guests on the part of our Eastern colleagues. They inform us that in addition to the routine for the conduct of ordinary business they have provided for showing strangers the city of Boston and an excursion by steamer down the Bay.

As this is the only meeting of the whole Association prior to the International one in Chicago in 1893, there is an urgent call for a full representation by all the members of the Association The Western representatives should turn out in force to assure us of a good representation at Chicago, and the Eastern representatives should appear in numbers in order not to weaken their own interest and to give a guarantee of their help at the next Western meeting. All the preliminary work for the International meeting should be completed at the one in Boston ; it commands a wide and broad consideration.

The decision of the amendment offered at Washington, relative to the qualifications which will be demanded of our future members, and the Association's attitude toward the Veterinary Colleges of North America in the length of their curriculi, will be considered. These are grave and important questions to solve; every side and feature of these questions should be heard. It demands a full representation from every Veterinary centre in our country.

Most important Committee Reports will be offered, and the all important question of our food supply will be considered. The position of our Association and the profession's status in regard to the milk and meat supply will be discussed.

From the pen of Dr. D. E. Salmon there will be a paper on the "Scientific Investigations of the Bureau of Animal Industry." Veterinary Science in Agricultural Colleges and Experiment Stations will be reviewed by Dr. W. L Williams, Purdue University. A summary of the field of original researches in the diseases of rodents, and many important discoveries will be given by Dr. S. E. Weber of Pennsylvania. "Strongylus Armatus" by Dr. J. T. Winchester of Massachusetts. Army Veterinary Practice, by Dr. D. Lemay of Kansas. A paper by Dr. Tait Butler of Mississippi, on "Professional Ethics," as related to business interests, and others of value are promised.

Application for the usual reduction in railroad rates has been made.

All State Associations should be prepared to be represented by duly appointed delegates.

R. S. Huidekoper, *President.*

W. Horace Hoskins, *Secretary.*

---

### NEW YORK STATE VETERINARY MEDICAL SOCIETY.

The New York State Veterinary Medical Society will hold its semi-annual meeting at the Vanderbilt House, Syracuse, on Thursday, July 14, 1892. The meeting commences 10 o'clock a. m. A cordial invitation is extended to every veterinarian in New York State who is a graduate of any veterinary college or university to attend this meeting, and be convinced that the object of this Society is to work for the protection of the qualified veterinarians in all portions of the State. No matter whether he be located in one of the largest cities or smallest towns, his professional interests are the same and need protection, which can only be obtained through the united efforts of all. It is earnestly requested that every qualified Veterinarian in the Empire State should attend this meeting, and prove to other members of the profession that he is willing and ready to assist them in advancing the standard of his profession. We must prove to the public that we are not to be classed with the "*farrier*" in a professional light. When this has been done we can command the attention of the Legislature in obtaining laws for the protection of the public at the hands of the Veterinarian. Lay aside all personal and professional business for one day and join us.

If you have any cases of interest in medicine or surgery that have come under your personal observation, the Society will be pleased to have you bring notes of the history and treatment of the same, to be read before its members for our mutual benefit.

Members of the profession who cannot attend and wish to become members of the Society, can do so by making application and sending the amount of annual dues ($2.00) and initiation fee ($5.00) by some member who will vouch for their credentials and professional standing. If accepted by the Board of Censors, his certificate of membership will be filled out and mailed to him.

N. P. HINKLEY, *Secretary.*

### Books and Pamphlets Received.

Die Accessorischen Geschlechtsdrusen der Sangethiere, von Dr. J. Sh. Oudemans. Haarlem, 1892.

Verhandlungen und Mittheinlungen des Siebenburgischen Vereins fur Naturwessenschaften in Hermannstadt, 1891.

Transactions of the Canadian Institute. No. 4, Vol. II, pt. 2. Toronto, 1892.

Annual Archæological Report and Canadian Institute Session, 1891. Toronto, 1891.

An Appeal to the Canadian Institute of the Rectification of Parliament, by Sandford Fleming, C. M. G.. LL. D. Toronto, 1892.

Journal and Proceedings of the Royal Society of New South Wales. Vol. XXV. Sydney, 1891.

Bulletin de L'Academie Royal de Medicine de Belgique, 1892.

Il Naturalista Siciliano. Palermo, 1892.

Johns Hopkins University Circulars. Baltimore.

Annalen des K. K. Naturhistorischen Hofmuseums. Redigirt von Dr. Franz Ritter von Hauer. Wein, 1891.

The Ottawa Naturalist.

Bollettino dei Musei di Zoologia ed Anatomia comparata della R. Universita di Torino. Vol. VII.

Fauna. Societe des Naturalistes Luxembourgeois, 1892.

Transactions of the Academy of Science of St. Louis. Vol. V, No. 3–4.

Appendix to the Catalogue of the Flora of Nebraska, by H. J. Weber. 1892.

The Canadian Record of Science. Vol. V, No. 1–2. Montreal.

Journal of the Bombay Natural History Society. Vol. VII, No. 4. Edited by H. M. Phipson.

Bulletin de la Societe Zoologique de France. Paris, 1892.

Bulletin Mensuel Societe Linneeune du Nord de la France. Amiens.

Abhandlungen von Naturwissenschaftlichen Vereine. Bremen, 1892.

